# A single large-scale mitochondrial DNA deletion presenting as rapidly progressive dementia in a 35-year-old male

**DOI:** 10.1038/s44400-026-00081-z

**Published:** 2026-04-27

**Authors:** Connor D. Dietz, Kaancan Deniz, Jamie C. Fong, Soonmee Cha, Katherine P. Rankin, Suzee E. Lee, Michael D. Geschwind, Charles C. Windon

**Affiliations:** 1 Edward and Pearl Fein Memory and Aging Center, Weill Institute for Neurosciences, Department of Neurology, University of California San Francisco, San Francisco, CA USA; 2 Department of Radiology and Biomedical Imaging, University of California San Francisco, San Francisco, CA USA

**Keywords:** Diseases, Neurology, Neuroscience

## Abstract

Single large-scale mitochondrial DNA deletions (SLSMDs) are among the most common mitochondrial disorders. Adult-onset rapidly progressive dementia (RPD) due to an SLSMD has not previously been described. A 35-year-old male was referred to our clinic for assessment of RPD after developing rapidly progressive cognitive, behavioral, and motor symptoms over 14 months. Serial brain MRIs demonstrated progressive severe left temporal, moderate right temporal, and mild global brain parenchymal atrophy without T2-weighted or diffusion-weighted MRI abnormalities. Blood and cerebrospinal fluid testing showed elevated nonspecific markers of neurodegeneration (neurofilament light chain, 14-3-3γ, and neuron-specific enolase). After excluding common etiologies of RPD, mitochondrial genome sequencing revealed a novel de novo SLSMD (m.608_14511del). Brain magnetic resonance spectroscopy of the left thalamus demonstrated spectra suggestive of a lactate peak. This case demonstrates that SLSMDs can present with RPD as the primary clinical manifestation and should be considered in the differential diagnosis of RPD in young adults.

## Introduction

Single large-scale mitochondrial DNA deletions (SLSMDs) are among the most common mitochondrial disorders, characterized by large deletions of the mitochondrial genome with variable heteroplasmy across tissues^1–3^. These deletions most often arise de novo, with an estimated prevalence of 1.2–1.6 per 100,000 individuals, and classically present with one of three clinical syndromes: Pearson syndrome (PS), Kearns–Sayre syndrome (KSS), or chronic progressive external ophthalmoplegia (CPEO)^4–6^.

PS is an infantile-onset disorder characterized by sideroblastic anemia and exocrine pancreatic dysfunction and is frequently fatal in infancy^1^. Patients who survive typically develop KSS, a multisystem disorder defined by onset before age 20, progressive external ophthalmoplegia, pigmentary retinopathy, and at least one of cardiac conduction block, cerebellar ataxia, or elevated cerebrospinal fluid (CSF) protein^2^. Additional features often include hearing loss, short stature, endocrinopathies (e.g., diabetes, hypoparathyroidism), and renal dysfunction^7^. CPEO is characterized by ptosis, ophthalmoplegia, and variable oropharyngeal and proximal limb muscle weakness^8^.

These syndromes are typically associated with SLSMDs ranging from approximately 1000 to 10,000 base pairs in length, with a common deletion size of 4977 base pairs^3,7^. Cognitive impairment and other neuropsychiatric symptoms have been described as part of their multisystem involvement but not as dominant or isolated presenting features^9^. Here, we describe the case of a 35-year-old male with a novel de novo SLSMD spanning base pairs m.608_14511, presenting with rapidly progressive dementia (RPD) as the primary clinical manifestation.

## Results

### Case presentation

The patient was a right-handed male with a normal prenatal history and birth. He had a developmental stutter and idiopathic toe walking until five years of age. At age five, he had a traumatic brain injury (TBI) due to a vehicle-on-pedestrian motor vehicle accident, resulting in a one-month intensive care unit admission. He fully recovered from this injury with no focal neurological deficits and graduated high school in the regular curriculum with average grades. After high school, he worked as a grocery store clerk, had a partner, and lived independently for more than 15 years before onset of his RPD.

His past medical history was notable for acetylcholine receptor (AChR) antibody–positive myasthenia gravis, diagnosed at age 31, which manifested as intermittent ptosis, diplopia, nasal speech, and dysphagia with solids (AChR binding antibody 3.10 nmol/L [reference ≤ 0.30 nmol/L], AChR modulating antibody 76% inhibition [reference < 32% inhibition], and AChR blocking antibody < 15% inhibition [reference < 15% inhibition]). These symptoms improved after treatment with pyridostigmine and palatoplasty surgery and resolved following a thymectomy.

With regard to family history, his 60-year-old mother had type 1 diabetes, Graves’ disease, and lifelong learning difficulties; his father died at age 43 from complications of alcohol use disorder; one of his brothers had congenital talipes equinovarus; his maternal grandmother died at age 81 from pancreatic cancer and was hospitalized for two months during adulthood for an unspecified psychiatric illness; his maternal grandfather died at age 82 from complications of diabetes; and his paternal grandparents died at ages 84 and 88 due to a stroke and lymphoma, respectively. There was no history of mitochondrial disease, dementia, parkinsonian disorders, amyotrophic lateral sclerosis, or other neurodevelopmental differences in his family. His parents were non-consanguineous.

At age 35, he developed rapid changes in his cognition and behavior, resulting in loss of functional independence within one month. His symptoms began with generalized anxiety, short-term episodic memory impairment, an aversion to foods with coarse textures, compulsive picking and checking behaviors, reduced spontaneous speech output, and visual hallucinations. After two months, he no longer recognized the faces of prior acquaintances, and by three months, he became profoundly apathetic and developed parkinsonism with flexion dystonia in his left hand. While his visual hallucinations gradually resolved, the other symptoms persisted, and when he was referred to our clinic for assessment at 14 months after symptom onset, neurological examination demonstrated bradyphrenia; hypomimia; hypokinetic dysarthria; and left arm rigidity, bradykinesia, flexion dystonia, and cortical sensory loss. There were no findings of external ophthalmoplegia, myopathy, peripheral neuropathy, or ataxia. He scored 18/30 on a Mini-Mental State Examination, missing points for orientation, delayed recall, and working memory. Further neuropsychology testing revealed severe impairments in verbal and visual memory; executive function; naming and repetition; and category, phonemic, and design fluency.

### Neuroimaging, laboratory, and genetic findings

A comprehensive diagnostic evaluation for RPD was performed using the VITAMINS mnemonic to systematically assess potential vascular, infectious, toxic–metabolic/mitochondrial, autoimmune/inflammatory, metastatic/neoplastic, iatrogenic, neurodegenerative, and systemic/seizure/structural etiologies^10–12^. The workup included neuroimaging (serial brain MRIs and magnetic resonance spectroscopy [MRS]), scrotal ultrasound, and contrast-enhanced CT of the chest, abdomen, and pelvis; laboratory studies of blood, urine, and CSF; a 72-hour ambulatory EEG; and whole nuclear and mitochondrial genome sequencing (Table 1). Whole nuclear and mitochondrial genome sequencing was performed on DNA extracted from a buccal swab using the GeneDx GenomeDx, Duo assay (Gaithersburg, MD, May 2025). Mitochondrial genome sequencing was pursued considering the patient’s maternal family history, including lifelong learning difficulties in his mother and an unspecified psychiatric illness requiring hospitalization in his maternal grandmother. MRS was performed after the results of the patient’s mitochondrial genome sequencing became available.

**Table 1:** Diagnostic workup completed for causes of rapidly progressive dementia

Etiologic Category	Tests	Abnormal Results
Vascular	Imaging: Brain MRI with contrast	-
Infectious	Blood: CBC and differential, HIV antigen and antibody, RPR, Lyme disease antibody Urine: Urinalysis and culture CSF: Cell count and differential, protein, glucose, HSV1/2 DNA, VDRL, bacterial gram stain and culture, AFB smear and culture, cryptococcal antigen, fungal culture Imaging: Brain MRI with contrast	-
Toxic-Metabolic/ Mitochondrial	Blood: CBC and differential, extended electrolytes, kidney function tests, liver function tests, glucose, TSH, free thyroxine, vitamin B_12_, folate, methylmalonic acid, homocysteine, thiamine, vitamin D, ammonia, lactate, ceruloplasmin, copper Urine: Urinalysis, 24-hour copper, random copper Imaging: Brain MRI, brain MRS Other: Mitochondrial genome sequencing^a^	Genetic testing identified a single large-scale mitochondrial DNA deletion (Fig. 2) Brain MRS showed a peak suggestive of lactate (Fig. 1B)
Autoimmune/ Inflammatory	Blood: Mayo Clinic Laboratories Autoimmune/Paraneoplastic Encephalopathy Evaluation (ENS2)^b^, oligoclonal banding, IgG index, CRP, ANA, extractable nuclear antigen antibodies, anti-dsDNA antibody, rheumatoid factor, MPO-ANCA, PR3-ANCA, anti-thyroglobulin antibody, anti-thyroid peroxidase antibody CSF: Cell count and differential, protein, glucose, oligoclonal banding, IgG index, Mayo Clinic Laboratories Autoimmune/Paraneoplastic Encephalopathy Evaluation (ENC2)^c^ Imaging: Brain MRI with contrast	ANA titer 1:80 nuclear, homogenous; ANA titer 1:160 nuclear, nucleolar (ref: < 1:40) Thyroid peroxidase antibody 132 IU/mL (ref: < 9 IU/mL) Thyroglobulin antibody 2 IU/mL (ref: ≤ 1 IU/mL)
Metastases/ Neoplastic	Blood: CBC and differential CSF: Cell count and differential, cytology, flow cytometry Imaging: Brain MRI with contrast; CT chest, abdomen, and pelvis with contrast; scrotal ultrasound	-
Iatrogenic	Urine: Drug screen Imaging: Brain MRI with contrast	-
Neurodegenerative	Blood: NfL^d^ CSF: National Prion Disease Pathology Surveillance Center RT-QuIC, total-tau, 14-3-3γ^e^; Mayo Clinic Laboratories Alzheimer’s disease evaluation (Aβ42, p-tau181, p-tau/Aβ42 ratio, total-tau) and NSE^f^ Imaging: Brain MRI Other: Whole nuclear genome sequencing^a^	Brain MRI showed atrophy (Fig. 1A) NfL 4.03 pg/mL (ref: 0.00-1.87 pg/mL) 14-3-3γ 3032 AU/mL (ref: 173-1999 AU/mL) NSE 21 ng/mL (ref: < 15 ng/mL)
Systemic/Seizures/ Structural	Imaging: Brain MRI with contrast Other: 72-hour ambulatory EEG	-

*Aβ42* Amyloid-β42, *AFB* Acid-Fast Bacilli, *ANA* Antinuclear Antibody, *ANCA* Anti-Neutrophil Cytoplasmic Antibody, *CBC* Complete Blood Count, *CRP* C-Reactive Protein, *dsDNA* Double-Stranded DNA, *HSV* Herpes Simplex Virus, *MPO* Myeloperoxidase, *MRS* Magnetic Resonance Spectroscopy, *NfL* Neurofilament Light Chain, *NSE* Neuron-Specific Enolase, *PR3* Proteinase 3, *RPR* Rapid Plasma Reagin, *RT-QuIC* Real-Time Quaking-Induced Conversion, *TSH* Thyroid-Stimulating Hormone, *VDRL* Venereal Disease Research Laboratory.

^a^ GeneDx GenomeDx, Duo, Buccal Swab (Gaithersburg, MD, May 2025).

^b^ Mayo Clinic Laboratories Encephalopathy, Autoimmune/Paraneoplastic Evaluation, Serum (Rochester, MN, March 2025).

^c^ Mayo Clinic Laboratories Encephalopathy, Autoimmune/Paraneoplastic Evaluation, Spinal Fluid (Rochester, MN, March 2025).

^d^ Labcorp Roche Diagnostics Electrochemiluminescence Immunoassay (ECLIA), Serum (Burlington, NC, March 2025).

^e^ National Prion Disease Pathology Surveillance Center Quantitative ELISA and RT-QuIC, Spinal Fluid (Cleveland, OH, March 2025).

^f^ Mayo Clinic Laboratories Neuron-Specific Enolase, Spinal Fluid (Rochester, MN, March 2025).

**Fig. 1:**
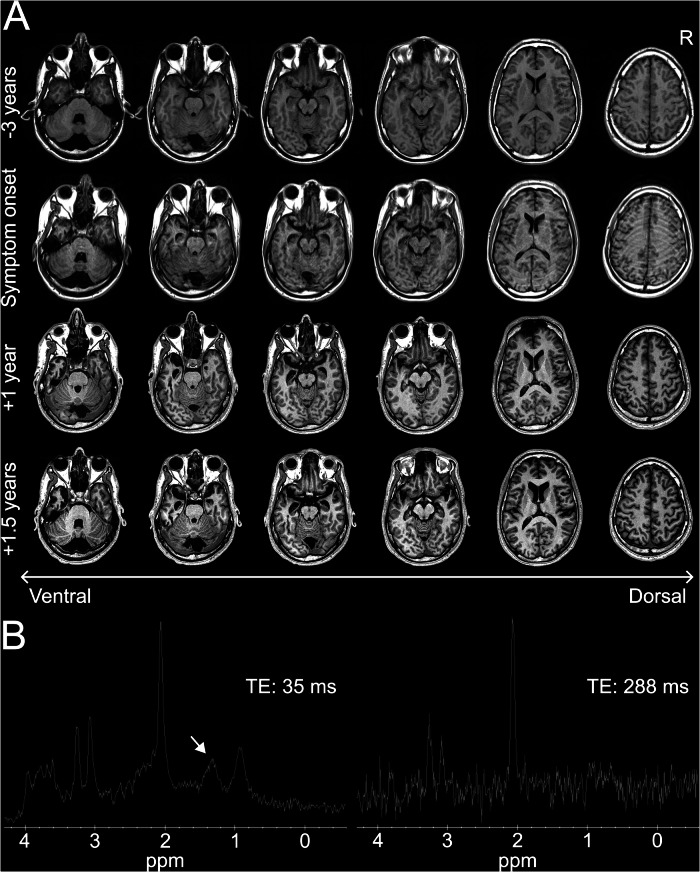
Longitudinal brain MRIs and proton magnetic resonance spectroscopy. **A** Unenhanced axial T1-weighted images ascending superiorly from left to right; each row corresponds to a different time point. Progressive severe left temporal, moderate right temporal, and mild global brain parenchymal atrophy with a subtle right frontoparietal, perirolandic predominance are seen over time. An area of chronic encephalomalacia at the left frontal pole and an arachnoid cyst posterior to the cerebellum are also visualized. **B** Single-voxel proton magnetic resonance spectroscopy centered in the left thalamus. Spectra from short (35 ms, left) and long (288 ms, right) echo times are presented. The solid white arrow highlights a broad peak centered around 1.3 ppm, suggestive of lactate. This peak is not reproduced when a long echo time is used. R right, TE echo time, ppm parts per million.

**Fig. 2:**
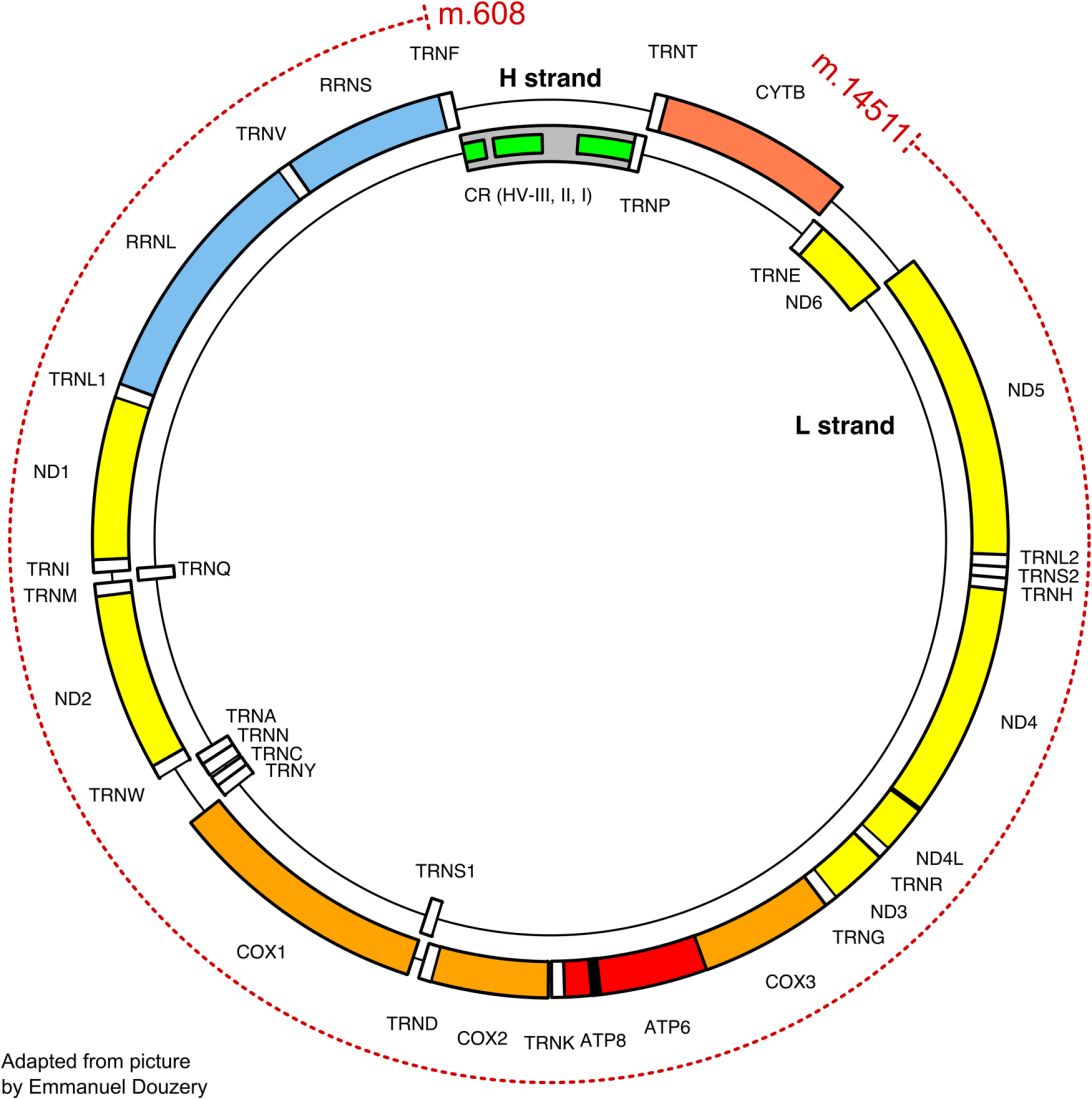
Illustration of the patient’s single large-scale mitochondrial DNA deletion. The red dashed line depicts the segment of mitochondrial DNA that was deleted, with a heteroplasmy level estimated at 5–15% on a buccal swab sample. The deletion spans base pairs 608–14,511, involving most of the 16,569 base pair mitochondrial genome. The 37 mitochondrial genes are indicated by their gene symbols. H strand = heavy strand, L strand = light strand, CR = control region, HV = hypervariable region. Adapted from an original image by Emmanuel Douzery (https://commons.wikimedia.org/wiki/File:Map_of_the_human_mitochondrial_genome.svg, Wikimedia Commons), licensed under CC BY-SA 4.0.

Serial brain MRIs demonstrated progressive severe left temporal, moderate right temporal, and mild global brain parenchymal atrophy with a subtle right frontoparietal, perirolandic predominance. In addition, an area of chronic encephalomalacia at the left frontal pole and an arachnoid cyst posterior to the cerebellum were identified (Fig. 1A). There were no abnormalities on T2-weighted or diffusion-weighted MRI. Blood and CSF testing showed elevations in nonspecific markers of neurodegeneration (neurofilament light chain [4.03 pg/mL, reference 0.00-1.87 pg/mL], 14-3-3γ [3032 AU/mL, reference 173-1999 AU/mL], and neuron-specific enolase [21 ng/mL, reference < 15 ng/mL]), a positive antinuclear antibody (titer 1:80 nuclear, homogenous; titer 1:160 nuclear, nucleolar; reference titer < 1:40), and elevated thyroid peroxidase and thyroglobulin antibodies (132 IU/mL, reference < 9 IU/mL and 2 IU/mL, reference ≤ 1 IU/mL, respectively; Table 1). There was no biochemical evidence of thyroid dysfunction (i.e., thyroid-stimulating hormone and free thyroxine levels were normal). CSF Alzheimer’s disease biomarkers were normal (Aβ42 893 pg/mL [reference > 834 pg/mL], p-tau181 10.8 pg/mL [reference ≤ 21.6 pg/mL], p-tau/Aβ42 ratio 0.012 [reference ≤ 0.028], and total-tau 140 pg/mL [reference ≤ 238 pg/mL]) and real-time quaking-induced conversion was negative for abnormal prion protein. Mitochondrial genome sequencing on DNA extracted from a buccal swab revealed the presence of an SLSMD (m.608_14511del) with a heteroplasmy estimated at 5-15% (Fig. 2). The patient’s mother was tested by Sanger sequencing and the deletion was not observed. Nuclear genome sequencing did not detect any pathogenic variants. Single-voxel proton MRS centered in the left thalamus demonstrated a broad peak around 1.3 ppm at a short echo time of 35 ms, suggestive of lactate. This probable lactate peak was not reproduced using a long echo time of 288 ms (Fig. 1B).

### Clinical management and course

Following the diagnosis of an SLSMD, the patient was prescribed a combination of supplements, including a B-vitamin complex, coenzyme Q10, vitamin E, and folinic acid, in accordance with current treatment guidelines for mitochondrial disease^13^. Additional targeted treatments for parkinsonism and dystonia were deferred in shared decision-making with the patient and his family in favor of monitoring the clinical response to the mitochondrial supplementation regimen. The patient’s clinical symptoms initially plateaued, followed by mild ongoing improvements in parkinsonism and dystonia. He has been followed longitudinally in our clinic for one year with multidisciplinary input from several subspecialties (ophthalmology, audiology, cardiology, gastroenterology, and medical genetics) to monitor for the development of medical comorbidities associated with mitochondrial disorders.

## Discussion

To our knowledge, this is the first reported case of an SLSMD manifesting as RPD in adulthood. Whereas other mitochondrial disorders, particularly mitochondrial encephalomyopathy with lactic acidosis and stroke-like episodes, have been found to present as RPD in adults, SLSMDs are not known to present in this manner^14,15^. Conversely, in more common causes of RPD such as sporadic Creutzfeldt-Jakob disease, deficiency of all mitochondrial respiratory chain complexes has been described, suggesting that mitochondrial dysfunction may play a broader role in RPD pathophysiology^16^.

Unlike the phenotypes classically associated with SLSMDs, our patient did not exhibit typical syndromic features such as external ophthalmoplegia, retinopathy, or endocrinopathies, although thyroid autoantibodies were present without biochemical evidence of thyroid dysfunction. This may be due to the patient’s low heteroplasmy level in peripheral tissues (5–15% on buccal swab), as SLSMD disease severity and age of onset have been shown to correlate with heteroplasmy levels^1^. Instead, his clinical course was dominated by rapid cognitive decline and behavioral symptoms, accompanied by parkinsonism, dystonia, and cortical sensory deficits, highlighting that SLSMDs can have atypical neurological presentations. Interestingly, in this case the patient’s motor symptoms and sensory deficits closely overlapped with those of corticobasal syndrome^17^.

The patient’s history is notable for a severe TBI in early life and autoimmune comorbidities, including AChR antibody–positive myasthenia gravis and thyroid autoantibodies. These factors may have contributed to disease expression by lowering brain reserve or increasing susceptibility to neurodegenerative processes^18,19^. Mitochondria play a central role in energy metabolism and immune regulation, and these coexisting stressors may have acted as “second hits” that unmasked underlying mitochondrial vulnerability^20,21^. His pattern of cerebral atrophy, with prominent involvement of metabolically demanding regions such as the temporal lobes and hippocampi, may reflect selective vulnerability of high-energy neural networks in the setting of impaired oxidative phosphorylation^22^.

While the estimated heteroplasmy level in the buccal swab sample was low (5–15%), heteroplasmy varies substantially between tissues, and low levels in peripheral samples do not exclude significant brain involvement. Post-mitotic tissues such as the central nervous system often harbor higher burdens of mutant mitochondrial DNA, which can drive a predominantly neurologic phenotype even in the absence of systemic findings^3,23,24^. The presence of a lactate peak on brain MRS provides additional evidence of impaired oxidative metabolism within the central nervous system^25,26^.

Taken together, this case expands the clinical spectrum of SLSMDs, illustrating that they can underlie RPD in the absence of classic syndromic features. It also underscores the diagnostic value of comprehensive genetic testing, including mitochondrial genome sequencing, in the workup of RPD when standard investigations are unrevealing.

## Methods

### Patient identification and assessment

The patient described in this case report was referred to the University of California, San Francisco Edward and Pearl Fein Memory and Aging Center clinic by a community neurologist for the evaluation of RPD. Two behavioral neurologists (C.D. and C.W.) conducted a retrospective chart review of the patient’s medical records to establish the clinical chronology and extract available neuroimaging and laboratory test results, performed clinical history taking and neurological examinations, arranged outstanding investigations, and prescribed treatments. Neuropsychology testing was conducted by a neuropsychologist (K.R.), and genetic counseling was provided by a licensed genetic counselor (J.F.). Clinical brain MRIs and MRS were interpreted by a neuroradiologist (S.C.).

### Ethical standards and consent for publication

The present study was conducted in accordance with the principles outlined in the Declaration of Helsinki. The patient’s legally authorized representative provided written informed consent for publication of this report.

## Supplementary information

Supplementary Information

## Data Availability

All relevant data are contained within the article. Data are derived from the patient’s medical records and are not publicly available due to privacy and ethical restrictions.
