# Supplementary material for: A single large-scale mitochondrial DNA deletion presenting as rapidly progressive dementia in a 35-year-old male
Source: NPJ Dement. 2026 Apr 27;2(1):25. doi: 10.1038/s44400-026-00081-z (PMC13121013; doi:10.1038/s44400-026-00081-z)
Supplement: Supplementary file 1 — Supplementary Information [file 44400_2026_81_MOESM1_ESM.docx]

**CARE Reporting Guidelines**

| Sections | Item Description | Location (or reason for not reporting) |
| --- | --- | --- |
| [1. Title](https://resources.equator-network.org/reporting-guidelines/care/items/title.html) | The area of focus and “case report” should appear in the title. | Title (manuscript title implies that the article is a case report) |
| [2. Keywords](https://resources.equator-network.org/reporting-guidelines/care/items/keywords.html) | The key elements of this case in 2–5 words. | Not included (not requested by the journal) |
| [3. Abstract](https://resources.equator-network.org/reporting-guidelines/care/items/abstract.html) | 3a – Introduction: What does this case add?  3b – Case presentation:   - The main symptoms of the patient(s). - The main clinical findings. - The main diagnoses and interventions. - The main outcomes.   3c – Conclusion: What are the main “take-away” lessons from this case? | Abstract |
| [4. Introduction](https://resources.equator-network.org/reporting-guidelines/care/items/introduction.html) | Brief background summary of the case referencing the relevant medical literature. | Introduction |
| [5a. Patient information](https://resources.equator-network.org/reporting-guidelines/care/items/patient-information.html) | 5a – Demographic information of the patient (age, gender, ethnicity, occupation).  5b – Main symptoms of the patient (chief complaint).  5c – Medical, family, and psychosocial history—including lifestyle and genetic information whenever possible, details about relevant comorbidities, and past interv… | Results, Case Presentation |
| [6. Clinical findings](https://resources.equator-network.org/reporting-guidelines/care/items/clinical-findings.html) | Describe the relevant physical examination (PE) findings. | Results, Case Presentation |
| [7. Timeline](https://resources.equator-network.org/reporting-guidelines/care/items/timeline.html) | Depict important date and times in this case (table or figure). | Results, Case Presentation  Figure 1 |
| [8. Diagnostic assessment](https://resources.equator-network.org/reporting-guidelines/care/items/diagnostic-assessment-and-diagnosis.html) | 8a – Diagnostic methods (e.g., physical examination, laboratory testing, imaging, questionnaires)  8b – Diagnostic challenges (e.g., financial, language, or cultural)  8c – Diagnostic reasoning including other diagnoses considered  8d – Prognostic characteristics (e.g., staging) where applicable. | Results, Case Presentation  Results; Neuroimaging, Laboratory, and Genetic Findings  Methods, Patient Identification and Assessment  Table 1 |
| [9. Therapeutic Intervention](https://resources.equator-network.org/reporting-guidelines/care/items/therapeutic-interventions.html) | 9a – Types of intervention (e.g., pharmacologic, surgical, preventive, self-care)  9b – Administration (e.g., dosage, strength, duration)  9c – Changes in intervention (with rationale). | Results, Clinical Management and Course |
| [10. Follow up and outcomes](https://resources.equator-network.org/reporting-guidelines/care/items/follow-up-and-outcomes.html) | 10a – Clinician and patient-assessed outcomes  10b – Important follow-up test results (positive and negative)  10c – Intervention adherence and tolerability (and how this was assessed)  10d – Adverse and unanticipated events. | Results, Case Presentation  Results; Neuroimaging, Laboratory, and Genetic Findings  Results, Clinical Management and Course |
| [11. Discussion](https://resources.equator-network.org/reporting-guidelines/care/items/discussion.html) | Discussion (including conclusion):  11a – Strengths and limitations of the management of this case  11b – Relevant medical literature  11c – Rationale for conclusions (including assessment of cause and effect)  11d – Main “take-away” lessons of this case report. | Discussion |
| [12. Patient perspective](https://resources.equator-network.org/reporting-guidelines/care/items/patient-perspective.html) | When appropriate patients should share their perspectives on the treatments they received. | Not included |
| [13. Informed consent](https://resources.equator-network.org/reporting-guidelines/care/items/informed-consent.html) | Did the patient give informed consent? Please provide if requested. | The patient’s legally authorized representative provided written informed consent for publication of this report |
